# *N*-(2-Chloro­phenyl­sulfon­yl)-2-methyl­propanamide

**DOI:** 10.1107/S1600536811004284

**Published:** 2011-02-09

**Authors:** K. Shakuntala, Sabine Foro, B. Thimme Gowda

**Affiliations:** a Department of Chemistry, Mangalore University, Mangalagangotri 574 199, Mangalore, India; b Institute of Materials Science, Darmstadt University of Technology, Petersenstrasse 23, D-64287 Darmstadt, Germany

## Abstract

In the title compound, C_10_H_12_ClNO_3_S, the amide H atom is *syn* with respect to the *ortho*-chloro group in the benzene ring and the C—S—N—C torsion angle is 64.35 (16)°. The benzene ring and the SO_2_—NH—CO—C segment form a dihedral angle of 87.4 (1)°. The crystal structure features inversion-related dimers linked by pairs of N—H⋯O hydrogen bonds.

## Related literature

For the sulfanilamide moiety in sulfonamide drugs, see; Maren (1976 ▶). For its ability to form hydrogen bonds in the solid state, see; Yang & Guillory (1972 ▶). For the hydrogen-bonding characteristics of sulfonamides, see; Adsmond & Grant (2001 ▶). For the effect of substituents on the crystal structures of sulfono­amides, see: Gowda *et al.* (2008 ▶, 2009 ▶, 2010 ▶)
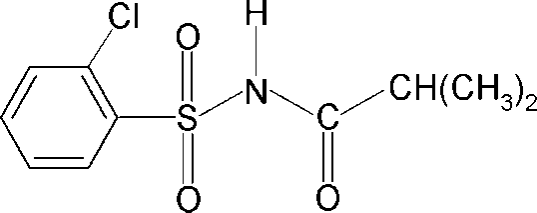


## Experimental

### 

#### Crystal data

C_10_H_12_ClNO_3_S
*M* *_r_* = 261.72
Triclinic, 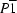

*a* = 8.365 (1) Å
*b* = 8.719 (1) Å
*c* = 9.143 (1) Å
α = 92.74 (1)°
β = 104.22 (1)°
γ = 108.75 (1)°
*V* = 606.24 (12) Å^3^
*Z* = 2
Mo *K*α radiation
μ = 0.48 mm^−1^
*T* = 293 K
0.45 × 0.35 × 0.35 mm

#### Data collection

Oxford Diffraction Xcalibur diffractometer with a Sapphire CCD detector
Absorption correction: multi-scan (*CrysAlis RED*; Oxford Diffraction, 2009 ▶) *T*_min_ = 0.814, *T*_max_ = 0.851
4031 measured reflections
2481 independent reflections
2200 reflections with *I* > 2σ(*I*)
*R*_int_ = 0.011

#### Refinement

*R*[*F* ^2^ > 2σ(*F* ^2^)] = 0.036
*wR*(*F* ^2^) = 0.102
*S* = 1.04
2481 reflections
149 parameters
1 restraint
H atoms treated by a mixture of independent and constrained refinement
Δρ_max_ = 0.39 e Å^−3^
Δρ_min_ = −0.27 e Å^−3^

### 

Data collection: *CrysAlis CCD* (Oxford Diffraction, 2009 ▶); cell refinement: *CrysAlis RED* (Oxford Diffraction, 2009 ▶); data reduction: *CrysAlis RED*; program(s) used to solve structure: *SHELXS97* (Sheldrick, 2008 ▶); program(s) used to refine structure: *SHELXL97* (Sheldrick, 2008 ▶); molecular graphics: *PLATON* (Spek, 2009 ▶); software used to prepare material for publication: *SHELXL97*.

## Supplementary Material

Crystal structure: contains datablocks I, global. DOI: 10.1107/S1600536811004284/ds2091sup1.cif

Structure factors: contains datablocks I. DOI: 10.1107/S1600536811004284/ds2091Isup2.hkl

Additional supplementary materials:  crystallographic information; 3D view; checkCIF report

## Figures and Tables

**Table 1:** Hydrogen-bond geometry (Å, °)

*D*—H⋯*A*	*D*—H	H⋯*A*	*D*⋯*A*	*D*—H⋯*A*
N1—H1*N*⋯O1^i^	0.84 (2)	2.14 (2)	2.976 (2)	174 (2)

Symmetry code: (i) 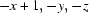.

**Fig. 1.**
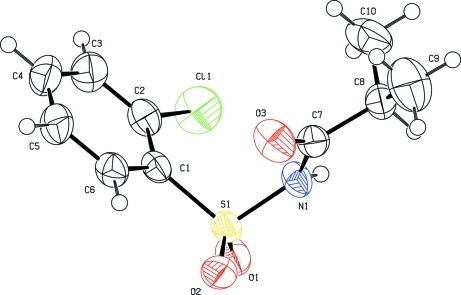
Molecular structure of the title compound, showing the atom- labelling scheme. Displacement ellipsoids are drawn at the 50% probability level.

**Fig. 2.**
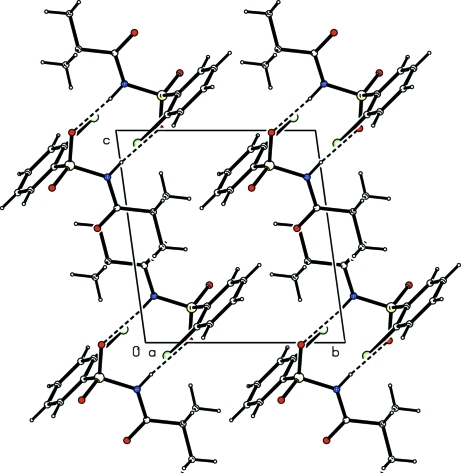
Molecular packing in the title compound. Hydrogen bonds are shown as dashed lines.

## supplementary crystallographic information

### Comment

The molecular structures of sulfonamide drugs contain the sulfanilamide moiety (Maren, 1976). The affinity for hydrogen bonding in the solid state due to the presence of various hydrogen bond donors and acceptors can give rise to polymorphism (Yang & Guillory, 1972). The hydrogen bonding preferences of sulfonamides has also been investigated (Adsmond & Grant, 2001). The nature and position of substituents play a significant role on the crystal structures of *N*-(aryl)sulfonoamides (Gowda *et al.*, 2008, 2009, 2010). As a part of studying the substituent effects on the structures of this class of compounds, the structure of *N*-(2-chlorophenylsulfonyl)-2,2-dimethylacetamide (I) has been determined. The conformations of the N—H and C=O bonds of the SO_2_—NH—CO—C segment in the structure are anti to each other (Fig. 1), similar to that observed in *N*-(phenylsulfonyl)-acetamide (II) (Gowda *et al.*, 2010), 2,2-dimethyl-*N*-(phenylsulfonyl)- acetamide (III)(Gowda *et al.*, 2009) and 2,2-dichloro-*N*- (phenylsulfonyl)-acetamide (IV) (Gowda *et al.*, 2008).

The molecule in (I) is bent at the *S*-atom with a C1—S1—N1—C7 torsion angle of 64.4 (2)°, compared to the values of -58.8 (4)° in (II), 67.1 (3)° in (III) and -66.3 (3)° in (IV). Further, the dihedral angle between the benzene ring and the SO2—NH—CO—C group in (I) is 87.4 (1)°, compared to the values of 89.0 (2)° in (II), 87.4 (1)° in (III) and 79.8 (1)° in (IV),

In the crystal structure, the intermolecular N–H···O hydrogen bonds (Table 1) link the molecules through inversion-related dimers into zigzag chains in the *bc*-plane. Part of the crystal structure is shown in Fig. 2.

### Experimental

The title compound was prepared by refluxing 2-chlorobenzenesulfonamide (0.10 mole) with an excess of 2,2-dimethylacetyl chloride (0.20 mole) for one hour on a water bath. The reaction mixture was cooled and poured into ice cold water. The resulting solid was separated, washed thoroughly with water and dissolved in warm dilute sodium hydrogen carbonate solution. The title compound was reprecipitated by acidifying the filtered solution with glacial acetic acid. It was filtered, dried and recrystallized from ethanol. The purity of the compound was checked by determining its melting point. It was further characterized by recording its infrared spectra.

Prism like colorless single crystals of the title compound used in X-ray diffraction studies were obtained from a slow evaporation of an ethanolic solution of the compound.

### Refinement

The H atom of the NH group was located in a difference map and later restrained to the distance N—H = 0.86 (2) Å. The other H atoms were positioned with idealized geometry using a riding model with C—H = 0.93–0.98 Å. All H atoms were refined with isotropic displacement parameters (set to 1.2 times of the *U*_eq_ of the parent atom).

### Crystal data

**Table:**

C_10_H_12_ClNO_3_S	*Z* = 2
*M**_r_* = 261.72	*F*(000) = 272
Triclinic, *P*1	*D*_x_ = 1.434 Mg m^−^^3^
Hall symbol: -P 1	Mo *K*α radiation, λ = 0.71073 Å
*a* = 8.365 (1) Å	Cell parameters from 2678 reflections
*b* = 8.719 (1) Å	θ = 3.0–27.7°
*c* = 9.143 (1) Å	µ = 0.48 mm^−^^1^
α = 92.74 (1)°	*T* = 293 K
β = 104.22 (1)°	Prism, colourless
γ = 108.75 (1)°	0.45 × 0.35 × 0.35 mm
*V* = 606.24 (12) Å^3^	

### Data collection

**Table:**

Oxford Diffraction Xcalibur diffractometer with a Sapphire CCD detector	2481 independent reflections
Radiation source: fine-focus sealed tube	2200 reflections with *I* > 2σ(*I*)
graphite	*R*_int_ = 0.011
Rotation method data acquisition using ω scans	θ_max_ = 26.4°, θ_min_ = 3.0°
Absorption correction: multi-scan (*CrysAlis RED*; Oxford Diffraction, 2009)	*h* = −10→10
*T*_min_ = 0.814, *T*_max_ = 0.851	*k* = −10→9
4031 measured reflections	*l* = −11→11

### Refinement

**Table:**

Refinement on *F*^2^	Secondary atom site location: difference Fourier map
Least-squares matrix: full	Hydrogen site location: inferred from neighbouring sites
*R*[*F*^2^ > 2σ(*F*^2^)] = 0.036	H atoms treated by a mixture of independent and constrained refinement
*wR*(*F*^2^) = 0.102	*w* = 1/[σ^2^(*F*_o_^2^) + (0.0589*P*)^2^ + 0.1796*P*] where *P* = (*F*_o_^2^ + 2*F*_c_^2^)/3
*S* = 1.04	(Δ/σ)_max_ < 0.001
2481 reflections	Δρ_max_ = 0.39 e Å^−^^3^
149 parameters	Δρ_min_ = −0.27 e Å^−^^3^
1 restraint	Extinction correction: *SHELXL97* (Sheldrick, 2008), Fc^*^=kFc[1+0.001xFc^2^λ^3^/sin(2θ)]^-1/4^
Primary atom site location: structure-invariant direct methods	Extinction coefficient: 0.074 (7)

### Special details

**Table:**

Experimental. CrysAlis RED (Oxford Diffraction, 2009) Empirical absorption correction using spherical harmonics, implemented in SCALE3 ABSPACK scaling algorithm.
Geometry. All e.s.d.'s (except the e.s.d. in the dihedral angle between two l.s. planes) are estimated using the full covariance matrix. The cell e.s.d.'s are taken into account individually in the estimation of e.s.d.'s in distances, angles and torsion angles; correlations between e.s.d.'s in cell parameters are only used when they are defined by crystal symmetry. An approximate (isotropic) treatment of cell e.s.d.'s is used for estimating e.s.d.'s involving l.s. planes.
Refinement. Refinement of *F*^2^ against ALL reflections. The weighted *R*-factor *wR* and goodness of fit *S* are based on *F*^2^, conventional *R*-factors *R* are based on *F*, with *F* set to zero for negative *F*^2^. The threshold expression of *F*^2^ > σ(*F*^2^) is used only for calculating *R*-factors(gt) *etc*. and is not relevant to the choice of reflections for refinement. *R*-factors based on *F*^2^ are statistically about twice as large as those based on *F*, and *R*- factors based on ALL data will be even larger.

### Fractional atomic coordinates and isotropic or equivalent isotropic displacement parameters (Å^2^)

**Table:**

	*x*	*y*	*z*	*U*_iso_*/*U*_eq_	
C1	0.8887 (2)	0.33504 (18)	0.17180 (18)	0.0374 (3)	
C2	0.9618 (2)	0.2693 (2)	0.07502 (19)	0.0455 (4)	
C3	1.1391 (3)	0.3366 (3)	0.0881 (2)	0.0571 (5)	
H3	1.1882	0.2924	0.0234	0.069*	
C4	1.2436 (3)	0.4688 (3)	0.1967 (3)	0.0581 (5)	
H4	1.3630	0.5137	0.2051	0.070*	
C5	1.1725 (3)	0.5347 (2)	0.2926 (2)	0.0546 (5)	
H5	1.2436	0.6243	0.3656	0.065*	
C6	0.9956 (2)	0.4682 (2)	0.2808 (2)	0.0441 (4)	
H6	0.9477	0.5128	0.3462	0.053*	
C7	0.7107 (2)	0.0482 (2)	0.36475 (18)	0.0397 (4)	
C8	0.6623 (2)	−0.1303 (2)	0.3835 (2)	0.0481 (4)	
H8	0.5375	−0.1848	0.3306	0.058*	
C9	0.6889 (5)	−0.1514 (4)	0.5501 (3)	0.0922 (9)	
H9A	0.6168	−0.1053	0.5915	0.111*	
H9B	0.8101	−0.0965	0.6046	0.111*	
H9C	0.6566	−0.2657	0.5599	0.111*	
C10	0.7668 (3)	−0.2058 (3)	0.3089 (3)	0.0720 (6)	
H10A	0.8900	−0.1503	0.3556	0.086*	
H10B	0.7401	−0.1958	0.2023	0.086*	
H10C	0.7368	−0.3194	0.3215	0.086*	
Cl1	0.83725 (8)	0.10188 (7)	−0.06290 (7)	0.0749 (2)	
N1	0.63339 (19)	0.07529 (16)	0.22009 (16)	0.0418 (3)	
H1N	0.578 (3)	−0.005 (2)	0.150 (2)	0.050*	
O1	0.55713 (17)	0.22202 (16)	0.00990 (15)	0.0571 (4)	
O2	0.63345 (17)	0.36011 (15)	0.27093 (17)	0.0567 (4)	
O3	0.8089 (2)	0.16099 (17)	0.46098 (15)	0.0609 (4)	
S1	0.66390 (5)	0.25610 (5)	0.16468 (5)	0.04147 (16)	

### Atomic displacement parameters (Å^2^)

**Table:**

	*U*^11^	*U*^22^	*U*^33^	*U*^12^	*U*^13^	*U*^23^
C1	0.0413 (8)	0.0280 (7)	0.0386 (8)	0.0104 (6)	0.0055 (6)	0.0046 (6)
C2	0.0544 (10)	0.0359 (8)	0.0409 (8)	0.0119 (7)	0.0098 (7)	−0.0009 (7)
C3	0.0622 (12)	0.0528 (11)	0.0604 (11)	0.0176 (9)	0.0277 (10)	0.0037 (9)
C4	0.0452 (10)	0.0501 (11)	0.0717 (13)	0.0050 (8)	0.0189 (9)	0.0054 (9)
C5	0.0481 (10)	0.0384 (9)	0.0610 (11)	0.0013 (8)	0.0072 (8)	−0.0068 (8)
C6	0.0469 (9)	0.0341 (8)	0.0452 (9)	0.0108 (7)	0.0077 (7)	−0.0024 (7)
C7	0.0393 (8)	0.0378 (8)	0.0400 (8)	0.0126 (7)	0.0090 (7)	0.0020 (6)
C8	0.0424 (9)	0.0400 (9)	0.0549 (10)	0.0088 (7)	0.0070 (8)	0.0130 (8)
C9	0.118 (2)	0.0910 (19)	0.0757 (17)	0.0331 (18)	0.0383 (16)	0.0458 (15)
C10	0.0806 (16)	0.0527 (12)	0.0824 (16)	0.0358 (12)	0.0069 (12)	−0.0016 (11)
Cl1	0.0767 (4)	0.0630 (4)	0.0670 (4)	0.0134 (3)	0.0098 (3)	−0.0306 (3)
N1	0.0444 (8)	0.0286 (7)	0.0412 (7)	0.0055 (6)	0.0017 (6)	0.0008 (5)
O1	0.0500 (7)	0.0446 (7)	0.0586 (8)	0.0094 (6)	−0.0090 (6)	0.0123 (6)
O2	0.0506 (7)	0.0399 (7)	0.0824 (10)	0.0184 (6)	0.0210 (7)	0.0004 (6)
O3	0.0738 (9)	0.0465 (7)	0.0459 (7)	0.0160 (7)	−0.0040 (6)	−0.0078 (6)
S1	0.0381 (2)	0.0302 (2)	0.0493 (3)	0.01005 (16)	0.00256 (17)	0.00424 (17)

### Geometric parameters (Å, °)

**Table:**

C1—C6	1.387 (2)	C7—C8	1.507 (2)
C1—C2	1.389 (2)	C8—C10	1.511 (3)
C1—S1	1.7659 (17)	C8—C9	1.514 (3)
C2—C3	1.380 (3)	C8—H8	0.9800
C2—Cl1	1.7344 (18)	C9—H9A	0.9600
C3—C4	1.377 (3)	C9—H9B	0.9600
C3—H3	0.9300	C9—H9C	0.9600
C4—C5	1.371 (3)	C10—H10A	0.9600
C4—H4	0.9300	C10—H10B	0.9600
C5—C6	1.379 (3)	C10—H10C	0.9600
C5—H5	0.9300	N1—S1	1.6396 (14)
C6—H6	0.9300	N1—H1N	0.843 (15)
C7—O3	1.208 (2)	O1—S1	1.4341 (13)
C7—N1	1.390 (2)	O2—S1	1.4202 (14)
			
C6—C1—C2	119.32 (16)	C7—C8—H8	108.1
C6—C1—S1	117.57 (13)	C10—C8—H8	108.1
C2—C1—S1	123.11 (13)	C9—C8—H8	108.1
C3—C2—C1	119.91 (16)	C8—C9—H9A	109.5
C3—C2—Cl1	118.08 (14)	C8—C9—H9B	109.5
C1—C2—Cl1	122.01 (14)	H9A—C9—H9B	109.5
C4—C3—C2	120.17 (18)	C8—C9—H9C	109.5
C4—C3—H3	119.9	H9A—C9—H9C	109.5
C2—C3—H3	119.9	H9B—C9—H9C	109.5
C5—C4—C3	120.34 (18)	C8—C10—H10A	109.5
C5—C4—H4	119.8	C8—C10—H10B	109.5
C3—C4—H4	119.8	H10A—C10—H10B	109.5
C4—C5—C6	119.98 (17)	C8—C10—H10C	109.5
C4—C5—H5	120.0	H10A—C10—H10C	109.5
C6—C5—H5	120.0	H10B—C10—H10C	109.5
C5—C6—C1	120.28 (17)	C7—N1—S1	124.59 (11)
C5—C6—H6	119.9	C7—N1—H1N	119.7 (15)
C1—C6—H6	119.9	S1—N1—H1N	115.1 (14)
O3—C7—N1	120.87 (16)	O2—S1—O1	118.79 (9)
O3—C7—C8	125.77 (16)	O2—S1—N1	109.66 (8)
N1—C7—C8	113.34 (14)	O1—S1—N1	104.14 (8)
C7—C8—C10	109.40 (16)	O2—S1—C1	107.71 (8)
C7—C8—C9	110.55 (18)	O1—S1—C1	110.42 (8)
C10—C8—C9	112.5 (2)	N1—S1—C1	105.30 (8)
			
C6—C1—C2—C3	0.1 (3)	O3—C7—C8—C9	23.2 (3)
S1—C1—C2—C3	−179.39 (15)	N1—C7—C8—C9	−158.19 (19)
C6—C1—C2—Cl1	179.41 (13)	O3—C7—N1—S1	−0.2 (2)
S1—C1—C2—Cl1	0.0 (2)	C8—C7—N1—S1	−178.89 (12)
C1—C2—C3—C4	−0.1 (3)	C7—N1—S1—O2	−51.29 (16)
Cl1—C2—C3—C4	−179.50 (16)	C7—N1—S1—O1	−179.42 (14)
C2—C3—C4—C5	0.0 (3)	C7—N1—S1—C1	64.35 (16)
C3—C4—C5—C6	0.2 (3)	C6—C1—S1—O2	4.95 (16)
C4—C5—C6—C1	−0.3 (3)	C2—C1—S1—O2	−175.59 (14)
C2—C1—C6—C5	0.1 (3)	C6—C1—S1—O1	136.13 (13)
S1—C1—C6—C5	179.61 (14)	C2—C1—S1—O1	−44.41 (16)
O3—C7—C8—C10	−101.1 (2)	C6—C1—S1—N1	−112.02 (14)
N1—C7—C8—C10	77.45 (19)	C2—C1—S1—N1	67.44 (15)

### Hydrogen-bond geometry (Å, °)

**Table:**

*D*—H···*A*	*D*—H	H···*A*	*D*···*A*	*D*—H···*A*
N1—H1N···O1^i^	0.84 (2)	2.14 (2)	2.976 (2)	174 (2)

Symmetry codes: (i) −*x*+1, −*y*, −*z*.
